# Oral Immunotherapy for Cow’s Milk Allergy: Five Years’ Experience from a Single Center in Turkey

**DOI:** 10.4274/balkanmedj.galenos.2020.2020.1.140

**Published:** 2020-10-23

**Authors:** Esen Demir, Nursen Ciğerci Günaydın, Figen Gülen, Remziye Tanaç

**Affiliations:** 1 Department of Pediatrics, Ege University School of Medicine, İzmir, Turkey; 2 Department of Pediatrics, Namık Kemal University School of Medicine, Tekirdağ, Turkey

**Keywords:** Children, cow’s milk allergy, oral immunotherapy

## Abstract

**Background:**

Oral immunotherapy for cow’s milk allergy is an effective treatment option because of its ability to increase the threshold for clinical reactions.

**Aims:**

To present our experience of oral immunotherapy for cow’s milk allergy in the pediatric allergy outpatient clinic, and to evaluate the long-term efficacy of oral immunotherapy and risk factors for adverse reactions during oral immunotherapy.

**Study Design:**

Single-center retrospective cohort study.

**Methods:**

Forty-two patients with Immunoglobulin-E-mediated cow’s milk allergy who complied with the oral immunotherapy protocol were evaluated in this study. The treatment consisted of a rapid escalation phase with an oral food challenge step that included milk doses. During the build-up phase, increasing quantities of cow’s milk were administered until the patient was able to consume 200 mL of cow’s milk daily.

**Results:**

The mean age of starting the oral immunotherapy was 40.2±3.2 (range, 36-156) months, and 54.8% (n=23) of the patients were males. The mean duration of the build-up phase was 18.1±5.6 (range, 9-41) weeks, and the mean maintenance phase was 29.1±11.6 (range, 12-63) months. During the oral immunotherapy, 36 adverse reactions (78% mild and 22% moderate) occurred in 16 (38%) patients. There were no differences in the age of starting the oral immunotherapy (p=0.19), cow’s milk-specific Immunoglobulin-E levels (p=0.17), and cumulative provocative doses of oral food challenges (p=0.78) between the two groups of patients with and without adverse reactions. The wheal diameters to cow’s milk were higher in the group with adverse reactions (p=0.03). There was no difference in the oral immunotherapy onset age between patients with and without a history of anaphylaxis (p=0.38). The patients with a history of anaphylaxis had more adverse reactions (p=0.04) and a higher number of reactions during the oral immunotherapy (p=0.01), and a higher mean duration of the up-dosing phase (p=0.04) compared with patients without anaphylaxis.

**Conclusion:**

Oral immunotherapy is a treatment option in patients with cow’s milk allergy because of its high efficacy. Adverse reactions occur in about 40% of cases and are mostly mild. It should be administered with caution to patients with a history of anaphylaxis and a higher wheal diameter to cow’s milk in the skin prick test.

Food allergy that causes serious reactions such as anaphylaxis or severe allergic reactions can be managed by allergen avoidance and symptomatic treatments (1). Children with food allergy and their families have reduced quality of life, especially if the allergy is severe (2). Cow’s milk allergy is the most common food allergy in infants and young children affecting 2%-3% of the latter population (3). The prevalence of food-challenge-defined allergy to cow’s milk (CM) was 0.6%-3% for all age groups (4). Eighty-seven percent of these patients will develop tolerance by 3 years of age (5). However, more recent studies have reported low rates of Cow’s milk allergy resolution (6,7). Skripak et al. (6) reported recovery rates of 19% by 4 years and 79% by 16 years of age.

Immunoglobulin-E (IgE)-mediated reactions due to CM intake may present as cutaneous reactions (e.g., urticaria, angioedema, and atopic dermatitis), respiratory reactions (asthma and rhinitis), gastrointestinal reactions (e.g., oral allergy syndrome and vomiting), or systemic reactions (anaphylaxis) (8). The current management of Cow’s milk allergy continues to be the avoidance of foods containing CM proteins until tolerance develops and the emergency treatment of acute reactions after accidental ingestions (9,10). However, CM can be present in a wide variety of foods, and strict avoidance is difficult, particularly in patients who react even to small amounts of CM. Allergen food avoidance leads to a poor quality of life for patients and their families because of the potential for unexpected sudden and life-threatening reactions (11). In various anaphylaxis series, Cow’s milk allergy accounted for 11%-28% of reactions, including up to 11% of fatal reactions (8).

To date, there is no effective pharmacologic agent that offers definitive treatment. Specific oral immunotherapy is a treatment option that has been introduced at several referral centers (12,13). It increases the threshold for clinical reactions if food tolerance is not achieved with age. The purpose of the oral immunotherapy is to protect against symptoms upon accidental ingestion, and to allow for full reintroduction of the food to the diet. Oral immunotherapy protocols generally start with a rapid escalation phase. In this phase, low amounts of CM are introduced, then it is rapidly increased to identify the maximum tolerated dose. Afterwards, the build-up phase follows, in which the daily dose is increased at weekly intervals until the target dose of 200 mL CM is attained. At the end of the build-up phase, the patient achieves desensitization, and continues to ingest 200 mL of CM regularly. The maintenance phase may continue for years. If the sustained unresponsiveness is to be evaluated, the patient avoids CM for a time period (generally 2-8 weeks) and then a new oral food challenge (OFC) is performed (14). Recently, the safety and efficacy results of long-term milk oral immunotherapy were investigated (15,16). In this study, we aimed to present our experience of oral immunotherapy for Cow’s milk allergy at a pediatric allergy outpatient clinic, to evaluate risk factors for the development of its adverse reactions, and to demonstrate its long-term effectiveness.

## MATERIALS AND METHODS

This study was a single-center retrospective cohort study. We performed power analysis to calculate the minimum number of patient to be included in the study. Approximately 3840 patients are examined in the child allergy outpatient clinic annually. The frequency of developing milk allergy in these patients is p=0.01. We calculated using 24 persons as the minimum number for the study with d=0.04 sampling error at 95% (α=0.05) confidence interval limits for a power of 0.8.

### Study population

### Patient selection

An oral immunotherapy protocol was administered to 47 patients aged 3-13 years who had only IgE-mediated Cow’s milk allergy aged between January 2009 and June 2014. The exclusion criteria of the oral immunotherapy were the unreliability of parents to manage the oral immunotherapy and concomitant non-controlled asthma.

### Study protocol

Of the 47 patients, 42 (89.3%) successfully complied with the protocol, one patient achieved partial tolerance, and 4 (8.5%) patients had a treatment failure. A 6-year-old female patient achieved only partial tolerance, tolerating the consumption of 45 mL CM once daily rather than the 200 mL. They should be considered as fully desensitized to a dose of 200 mL CM intake daily. Three patients withdrew from the protocol due to mild or moderate adverse effects, and their families were non-compliant with the treatment by not giving the prescribed doses at home. Another 3-year-old with high CM-specific IgE (sIgE) (>100) levels and a wheal diameter of 16 mm in the skin prick test (SPT) was forced to withdraw due to the development of moderate and severe reactions. She developed generalized urticaria with 3 mL CM and moderate bronchospasm with 6 mL CM in the OFC.

In this study, we evaluated 42 patients who could tolerate 200 mL milk at the end of the oral immunotherapy. All the patients were evaluated with an SPT and serum CM-sIgE antibodies for the diagnosis of Cow’s milk allergy. Additionally, an open OFC was performed in all the patients, except in those who had a recent anaphylaxis with CM.

### Skin prick test

The skin prick tests were performed using ALK-Abello A/S, Horsholm, Denmark standard prick test solutions (cow milk). The positive control was histamine and the negative control was 0.9% sodium chloride. The wheal diameters that were 3 mm and above according to the negative control were considered as positive.

### Laboratory evaluation

The total serum IgE and milk-sIgE levels were measured using the CAP system-FEIA (Pharmacia Upjohn). The detection limit was 0.35 kUA/l for sIgE.

### Oral food challenge test

The OFC test was an open protocol. OFCs were started using 0.1 mL diluted pasteurized CM with 3.3% protein content (1:10, milk:water), and were continued with increasing amounts of milk as follows: 0.1 mL, 1 mL, 2 mL, 3 mL diluted CM; and 1 mL, 1.5 mL, 3 mL, 6 mL, 12 mL, 24 mL, 50 mL, and 100 mL of undiluted CM until a reaction was noted. The oral challenge results were considered positive when objective symptoms occurred. Early and late objective reactions such as urticaria, angioedema, airway obstruction signs (e.g., dyspnea, rales, and rhonchi), vomiting, and anaphylaxis were assessed. We did not stop the OFCs for subjective reactions such as pruritus, not feeling well, and abdominal pain that were reported, which would increase the risk of false-positive test results when the reaction improved spontaneously in 10-20 minutes. We identified the individual tolerated doses of CM during the increased-dose OFCs. We performed the OFC test at 6-month or one-year intervals to evaluate the development of tolerance.

### Milk oral immunotherapy protocol

Persistent Cow’s milk allergy was evaluated according to the following criteria 4 weeks before the oral immunotherapy: The CM wheal diameter in SPT ≥3 mm and CM-sIgE >0.35 kUA/l for whole CM. After SPT and sIgE measurement, we performed the OFC tests. If the Cow’s milk allergy in the patients persisted, we informed the patients and their families about oral immunotherapy and allowed them to make an informed decision about the therapy.

If the patient decided to be treated with the oral immunotherapy, we accepted the OFC steps as part of an initial escalation phase on the first day. We evaluated the dose in the previous step before developing the reaction in the OFC using the tolerated dose (the dose steps in the OFC were as follows: 0.1 mL, 1 mL, 2 mL, 3 mL diluted CM; and 1 mL, 1.5 mL, 3, 6, 12, 24, 50, and 100 mL undiluted CM). On the second day, we continued the oral immunotherapy with a dose three steps behind the tolerated dose of the OFC on the first day, different from the other protocols. The patient was instructed to consume the same dose daily at home following week. During the build-up phase, increasing quantities of CM were administered initially at the hospital. If no reaction occurred, the same dose was continued at home weekly until the patient was able to consume the target dose of 200 mL (6540 mg protein) of CM daily at the end of 16 weeks (Table 1). During the home administrations, patients were able to contact the physician on their mobile phones 24 hours per day. The CM dose was modified during the follow-up period, according to the patient’s adverse events. When a dose in the build-up phase was not tolerated, the patient received the previous tolerated dose for one week at home. Patients were treated with antihistamines (1 mg ketotifen) once a day throughout the build-up phase. This phase was longer in patients who experienced adverse reactions. At the end of the build-up phase, the patients had achieved desensitization, and continued daily milk consumption during a maintenance phase. During the maintenance phase of the oral immunotherapy, the patients’ antihistamines treatment was discontinued.

Family members were instructed on the recognition of the adverse effects. Carrying an epinephrine auto-injector was advised in case of severe adverse effects, and both the patients and their families were instructed on their use. Adverse events were recorded as standard at each weekly visit. Adverse reactions were assessed and classified according to severity as mild (oral allergy syndrome, localized erythema or urticaria, vomiting, rhinitis, conjunctivitis, local urticarial, vomiting), moderate (generalized urticaria and angioedema, mild bronchospasm), and severe (moderate/severe bronchospasm, shortness of breath, breathing difficulties with inspiratory stridor, anaphylactic shock) (17). The patients were examined for 1-3 years to measure milk-sIgE. The study was approved by the local ethics committee (IRB no. 14-4.1/16) and informed consent was obtained from all the parents/guardians.

### Statistical analysis

Statistical analyses were performed using IBM SPSS Statistics V25. Descriptive statistics were expressed as mean, standard deviation, median, minimum and maximum values, frequencies, and percentages. Whether the distribution of each variable in the dataset fits the normal distribution was tested and variables that were not normally distributed were evaluated by non-parametric tests. A chi-square test was used in the analysis of the categorical data. Mann-Whitney U was used in binary independent group comparisons. The Spearman Rho correlation analysis was performed to assess the correlation between the scale scores. Friedman analysis was used to compare repeated mesurements and Dunn’s post-hoc test was used for binary comparisons when the significance was achieved. P<0.05 was considered statistically significant.

## RESULTS

The mean age of the patients was 40.2±3.2 (range, 36-156) months at the start of the oral immunotherapy, and 54.8% (n=23) of the patients were males. Seven percent (n=3) of patients were older than 5 years of age. The symptoms at presentation within the first 2 hours of milk ingestion were skin eruption (urticaria and/or angioedema) (83.3%, n=35), respiratory distress (23.8%, n=10), and vomiting (21.4%, n=9). Concomitant diseases were atopic dermatitis (45%, n=19), asthma (12%, n=5), and allergic rhinitis (10.5%, n=4). Seven (16.7%) patients had a history of anaphylaxis after exposure to CM. Eighty five percent of the patients presented with only cutaneous symptoms. Ten of 42 patients had CM-sIgE greater than 50 kUA/l. Table 2 shows the demographic characteristics and laboratory findings of the patients.

While there was no correlation between the laboratory findings and the duration of the build-up phase of treatment and the oral immunotherapy onset age of the patients; there was a correlation between CM-sIgE and IgE levels, and CM-sIgE levels were also high in patients with large CM wheal diameters in the SPTs (Table 3).

All the patients could not tolerate baked milk. In the OFC, the reactions were generally observed in response to the non-diluted milk dose. The median cumulative provocative dose in the OFC was 6 (0.2-48) mL. The provocation reactions consisted of urticaria (83.3%), respiratory distress findings (including bronchospasm, wheezing, and/or sibilant rhonchi) (7.1%), angioedema (12%), and vomiting (2.3%).

The mean duration of the build-up phase was 18.1±5.6 (range, 9-41) weeks. The mean duration of the maintenance phase was 29.1±11.6 (range, 12-63) months. During the oral immunotherapy, 36 adverse reactions occurred in 16 (38%) patients; 28 adverse reactions were mild and 8 were moderate. These adverse reactions consisted of localized urticaria (47%, n=17), respiratory distress (cough and bronchospasm; n=4 or cough and wheezing; n=3) (19.4%, n=7), gastrointestinal symptoms (vomiting, 11%, n=4), exacerbation of atopic dermatitis (8%, n=3), localized erythema and itching of the throat (8%, n=3), urticaria and angioedema (2.7%, n=1), and generalized urticaria (2.7%, n=1) (Table 4). All the reactions occurred in the hospital. The reactions were seen during the first 4 weeks in 50% of patients during the build-up phase. The mean reaction time was 5.2±3.5 (range, 1-14) weeks. Urticaria was controlled using oral antihistamines, and respiratory distress using oral antihistamines, steroids, and inhaled β2-agonists. No medications were administered for vomiting. We admitted patients’ reactions as single system involvement (only cutaneous or respiratory or gastrointestinal system signs) and after the treatment of the reactions, the patients did not develop any more symptom requiring the use of epinephrine.

Between the two groups of patients with and without adverse reactions, there were no differences in the oral immunotherapy onset age of the patients (p=0.19), CM-sIgE levels (p=0.17), and cumulative provocative doses of OFCs (p=0.78). The wheal diameters to CM were smaller (p=0.03) and the mean duration of the build-up phase was shorter (p<0.01) in the group without adverse reactions (Table 5).

Sex distribution (47%, 52.6% of the patients were females, respectively, p=0.26) and presence of additional atopic disease (40%, 60% of the patients, respectively, p=0.75) both were similar in patients with and without oral immunotherapy adverse reactions.

There was no difference in oral immunotherapy onset age between patients with and without a history of anaphylaxis (p=0.38). Also, patients with anaphylaxis had higher median IgE (p=0.02) and CM-sIgE (p=0.006), larger wheal diameters to CM in SPTs (p=0.001), higher cumulative provocative doses of OFCs (p=0.06, NS), more adverse reactions (p=0.04) and a higher number of reactions during the oral immunotherapy (p=0.01), and longer mean duration of the build-up phase (p=0.03) compared with patients without anaphylaxis (Table 6). Sex distribution was similar in patients with and without a history of anaphylaxis (71.4%, 51.4% of the patients were males, respectively, p=0.42).

**Figure 1:**
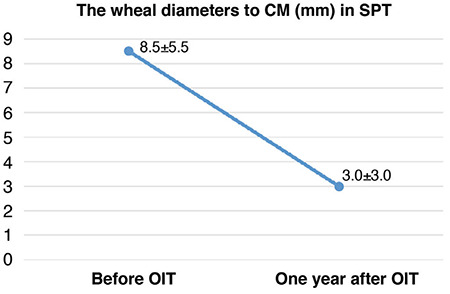
Wheal diameters (mm) to cow’s milk (commercial extract) in the skin prick test. CM: cow’s milk, SPT: skin prick test, OIT: oral immunotherapy

**Figure 2:**
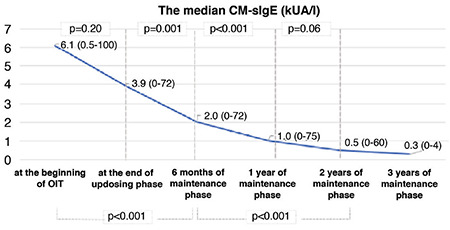
Median CM-sIgE level (kUA/l) results in the three years follow-up OIT. CM: cow’s milk, sIgE: specific immunoglobulin-E, OIT: oral immunotherapy

After one year of the maintenance phase, wheal diameters with CM in SPTs were significantly decreased (Figure 1). The median CM-sIgE levels decreased significantly step-by-step after the 6^th^ month, and one, two, and three years of the maintenance phase (Figure 2).

After completing the oral immunotherapys, the nutritional statuses of the patient were normal and none of them developed eosinophilic esophagitis.

## DISCUSSION

The treatment of Cow’s milk allergy is the dietary elimination of the CM protein. The dietary elimination is a difficult approach due to the possible risk of reactions, including anaphylactic reaction, following the accidental ingestion of CM. Oral immunotherapy is the active treatment for IgE-mediated CM allergic children, which is a suitable treatment compared to avoidance. Successful CM-oral immunotherapy protocols in different age groups have been reported. In some desensitization protocols, due to the probability that natural tolerance will develop in children by the age of 4 years, children older than 4 years have been treated with oral immunotherapy (12,13,18,19). However, more recent studies reported lower rates of natural tolerance development. In the studies by Garcia-Ara et al. (7), 68% of patients with Cow’s milk allergy developed tolerance by the age of 4 years. Other studies on oral immunotherapy at an earlier age have been published, such as those by Martorell et al. (20) (median age 2 years) and Staden et al. (21) (median age 2.5 years). The mean age of the patients receiving oral immunotherapy was 40.2±3.2 months in this study.

Most patients with Cow’s milk allergy are successfully desensitized, and many effective protocols have been described (13,20,21). In the protocol by Zapatero et al. (13), treatment began on day 1 with diluted doses; and on day 2, patients received a single dose of 1 mL non-diluted milk, and then a 2 mL dose 30 min later at the hospital, continuing with the same dose at home for a week. The dose of milk was increased once a week, until the patient was able to tolerate 200-250 mL non-diluted milk without having reactions (13). Meglio et al. (19) successfully desensitized 71.4% (n=15) of their patients and partially desensitized 14.3% (n=3) using a 6-month oral immunotherapy protocol. Their protocol started with one drop of diluted CM, increasing to 200 mL over a period of several months (19). In the study by Patriarca et al. (22), desensitization was started with one drop of CM and continued over a period of 136 days until the patient tolerated 120 mL CM.

In our protocol, the initial dose was dependent on the last tolerated dose in the OFC. Due to variable initial doses and development of reactions, the duration of the build-up phase varied from 9 to 41 weeks.

In the group of children with CM-sIgE antibody levels ≥50 kUA/L evaluated by Skripak et al. (6), some children outgrew their allergy during adolescence. In our study, 10 of 42 patients had CM-sIgE levels ≥50 kUA/L [74.7±21 (51.7-100)]. In some studies, immunotherapy was associated with a decrease in the CM- sIgE levels (8,9,20), whereas other studies found no difference in the levels of these antibodies (12,23). In our patients, CM-sIgE levels steadily decreased during the 3 years of follow-up after the oral immunotherapy. As in other studies, wheal diameters to CM decreased after 1 year (18).

The reported rates of adverse reactions in patients undergoing oral immunotherapy are in the range of 47-100% of patients (12,20,21,24,25). Lower ratios were reported by Meglio et al. (19) (61%) and Patriarca et al. (22) (51.5%). Adverse reactions are mainly mild or moderate and develop independently of desensitization regimens (21,23,25), even though the oral immunotherapy was associated with an increased risk of severe adverse reactions requiring adrenaline injection or systemic corticosteroids compared with those on an elimination diet (26). Some studies reported higher rates of adverse reactions requiring adrenaline injections (12,20,23,24). In the present study, 38% (n=16) of the patients developed adverse events on 36 occasions; 28 of the adverse reactions were mild and 8 were moderate. No severe reactions developed and there was no need to use adrenaline. In the study by Staden et al. (21), all the patients had mild reactions and four patients had moderate reactions. Adverse reactions were more frequent in the initial phases, especially during the first 4 weeks (50% of patients), as reported by Martorell et al. (20).

Before to the oral immunotherapy, our patients were premedicated with ketotifen during the build-up phase, whereas cetirizine or sodium cromoglycate was used in other protocols (19,22,27). Jagdis et al. (28) showed that ketotifen premedication reduced the frequency and severity of gastrointestinal adverse reactions during peanut oral immunotherapy. In our study, gastrointestinal adverse reactions were observed in 11% (n=4) of the patients, although they were seen in 2.3% (n=1) of patients in the OFC before the oral immunotherapy. In our experience, antihistamines did not mask any symptoms, except oral allergy syndrome or rash, and facilitated patient compliance as reported by Sanchez-Garcia et al. (29). Ketotifen was stopped after the up-dosing phase of treatment, and CM desensitization persisted.

Rates of successful desensitization to CM-oral immunotherapy were reported as 66.6%-90% (13,19,20,23), although Staden et al. (21) reported lower permanent tolerance rates (36%) after oral immunotherapy.

Our oral immunotherapy protocol had a 91.3% (n=42) success rate in achieving the target dose of 200 mL of CM daily. Additionally, 2% (n=1) of patients achieved partial desensitization with 45 mL of CM per day. The success of the treatment may be related to the patient characteristics. However, some protocols excluded patients with a history of anaphylaxis before oral immunotherapy (20), Alvaro et al. (30) in their study showed that oral desensitization to CM was efficient even in patients with anaphylactic reactions to CM in a one-year assessment. In this study, successful completion of treatment by patients with a history of anaphylaxis revealed the effectiveness of the oral immunotherapy protocol in our patients in the three years follow-up of the oral immunotherapy. Oral immunotherapy trials on patients with anaphylaxis are needed to investigate its safety. This study showed that adverse reactions and number of reactions during oral immunotherapy were higher in patients with a history of anaphylaxis.

There is insufficient evidence as to whether clinical tolerance is transient or persistent (31). After stopping the oral immunotherapy, patients may fail to achieve desensitization (32,33). In Sato et al.’s (31) study, the clinical tolerance ratio was 27.1% at the end of the milk oral immunotherapy. Therefore, we cannot estimate the loss of tolerance when ingestion is discontinued, which was reported by some studies (33). We could not evaluate the sustained unresponsiveness rates.

An open OFC protocol was chosen, although the patients were older than 3 years of age. We did not evaluate the isolated CM proteins (casein, a-lactoalbumin, ß-lactoglobulin) to assess persistence of CM allergy in patients and any parameter except SPT and CM-sIgE were not evaluated after oral immunotherapy either. We also did not perform any implementation for the evaluation of sustained unresponsiveness and permanent tolerance.

Oral immunotherapy is a promising treatment option for patients with Cow’s milk allergy because of its high efficacy. However, it is a time-consuming treatment and bears the risk of adverse reactions. Due to adverse reactions, it should be applied carefully to patients with higher CM wheal diameters in SPT. It can only be performed by experienced allergy departments and requires patient and family adaptation. However, there are limited data on their safety and long-term clinical follow-up. We successfully administered oral immunotherapy to children with or without a history of CM-related anaphylaxis. In this study, we describe our oral immunotherapy experience and the long-term results show that it is an effective treatment option for children with CM allergy. Despite the difficulties of oral immunotherapy, it can provide a better quality of life for patients and their families. Therefore, in experienced centers, with careful monitoring of patients, oral immunotherapy can be a safe treatment option, even in patients with a history of anaphylaxis.

## Figures and Tables

**Table 1:**
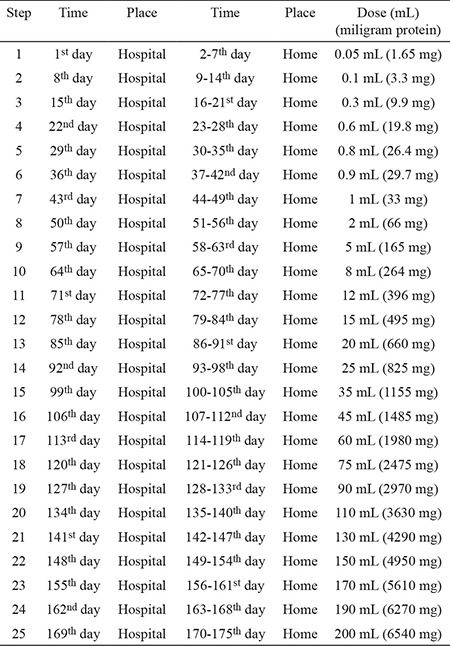
Oral immunotherapy protocol for cow’s milk

**Table 2:**
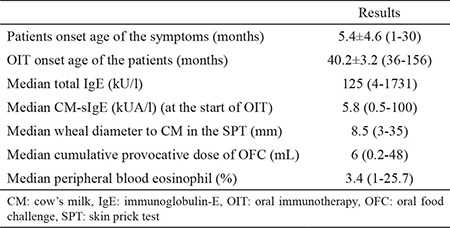
Demographic characteristics and laboratory findings

**Table 3:**
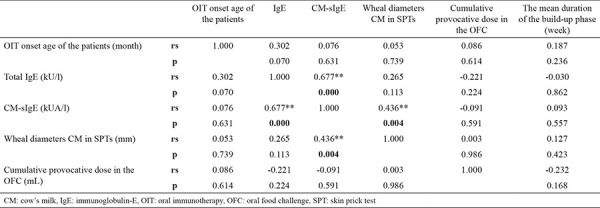
Evaluation of the correlation between laboratory findings, the mean duration of the up-dosing phase, and OIT onset age of the patients

**Table 4:**
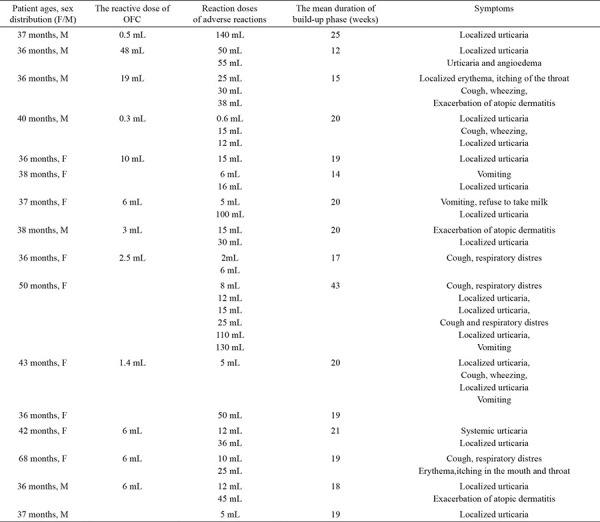
Characteristics of the adverse reactions of the patient

**Table 5:**
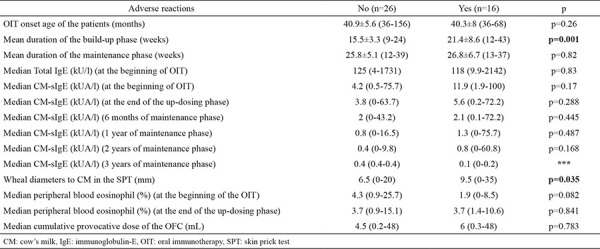
Oral immunotherapy duration and laboratory findings between the two groups of patients with and without adverse reactions

**Table 6:**
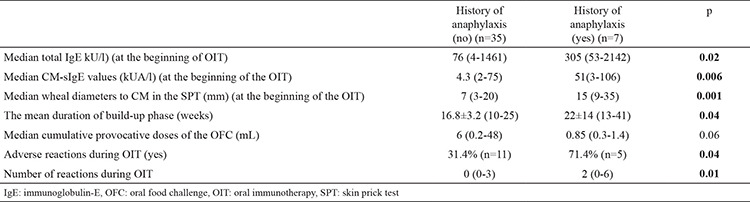
Patient characteristics according to the history of anaphylaxis

## References

[1] Boyce JA, Assa’ad A, Burks AW, Jones SM, Sampson HA, Wood RA, et al (2010). Guidelines for the diagnosis and management of food allergy in the United States: Summary of the NIAID-Sponsored Expert Panel. J Allergy Clin Immunol.

[2] Flokstra-de Blok BM, DunnGalvin A, Vlieg-Boerstra BJ, Oude Elberink JN, Duiverman EJ, Hourihane JO, et al (2008). Development and validation of the self-administered Food Allergy Quality of Life Questionnaire for adolescents. J Allergy Clin Immunol.

[3] Host A (2002). Frequency of cow's milk allergy in childhood. Ann Allergy Asthma Immunol.

[4] Nwaru BI, Hickstein L, Panesar SS, Roberts G, Muraro A, Sheikh A (2014). EAACI Food Allergy and Anaphylaxis Guidelines Group. Prevalence of common food allergies in Europe: a systematic review and meta-analysis. Allergy.

[5] Host A, Halken S (1990). A prospective study of cow milk allergy in Danish infants during the first 3 years of life: clinical course in relation to clinical and immunological type of hypersensitivity reaction. Allergy.

[6] Skripak JM, Matsui EC, Mudd K, Wood RA (2007). The natural history of IgE-mediated cow’s milk allergy. J Allergy Clin Immunol.

[7] Garcia-Ara MC, Boyano-Martinez MT, Diaz-Pena JM, Martin-Munoz MF, Martin-Esteban M (2004). Cow’s milk-specific immunoglobulin E levels as predictors of clinical reactivity in the follow-up of the cow’s milk allergy infants. Clin Exp Allergy.

[8] Fiocchi A, Brozek J, Schünemann H, Bahne SL, Von Berg A, Beyer K, et al (2010). World Allergy Organization (WAO) Diagnosis and Rationale for Action against Cow’s Milk Allergy (DRACMA) Guidelines. Pediatr Allergy Immunol.

[9] Wang J, Sampson HA (2011). Food allergy. J Clin Invest.

[10] Nowak-Wegrzyn A, Sampson HA (2011). Future therapies for food allergies. J Allergy Clin Immunol.

[11] Cohen BL, Noone S, Munoz-Furlong A, Sicherer SH (2004). Development of a questionnaire to measure quality of life in families with a child with food allergy. J Allergy Clin Immunol.

[12] Skripak JM, Nash SD, Rowley H, Brereton NH, Oh S, Hamilton RG, et al (2008). A randomized, double-blind, placebo-controlled study of milk oral immunotherapy for cow’s milk allergy. J Allergy Clin Immunol.

[13] Zapatero L, Alonso E, Fuentes V, Martínez MI (2008). Oral desensitization in children with cow's milk allergy. J Investig Allergol Clin Immunol.

[14] Feuille E, Nowak-Węgrzyn A (2016). Oral Immunotherapy for food allergies. Ann Nutr Metab.

[15] Kauppila TK, Paassilta M, Kukkonen AK, Kuitunen M, Pelkonen AS, Makela MJ (2019). Outcome of oral immunotherapy for persistent cow's milk allergy from 11 years of experience in Finland. Pediatr Allergy Immunol.

[16] Alves-Correia M, Gaspar A, Borrego LM, Azeyedo J, Martins C, Morais-Almeida M (2019). Successful oral desensitization in children with cow's milk anaphylaxis: Clinical and laboratory evaluation up to nine-years follow-up. Allergol Immunopathol (Madr).

[17] Clark AT, Ewan PW (2003). Food allergy in childhood. Arch Dis Child.

[18] Vázquez-Ortiz M, Alvaro-Lozano M, Alsina L, Garcia-Paba MB, Piquer-Gilbert M, Giner-Munoz MT, et al (2013). Safety and predictors of adverse events during oral immunotherapy for milk allergy: severity of reaction at oral challenge, specific IgE and prick test. Clin Exp Allergy.

[19] Meglio P, Bartone E, Plantamura M, Arabito E, Giampietro PG (2004). A protocol for oral desensitization in children with IgE-mediated cow's milk allergy. Allergy.

[20] Martorell A, De la Hoz B, Ibanez MD, Bone J, Terratos MS, Michauila A, et al (2011). Oral desensitization as a useful treatment in 2-year-old children with cow’s milk allergy. Clin Exp Allergy.

[21] Staden U, Rolinck-Werninghaus C, Brewe F, Wahn U, Niggemann B, Beyer K (2007). Specific oral tolerance induction in food allergy in children: efficacy and clinical patterns of reaction. Allergy.

[22] Patriarca G, Nucera E, Roncallo C, Pollastrini E, Bartolozzi F, De Paquale T (2003). Oral desensitizing treatment in food allergy: clinical and immunological results. Aliment Pharmacol Ther.

[23] Pajno GB, Caminiti L, Ruggeri P, De Luca R, Vita D, La Rosa M, et al (2010). Oral immunotherapy for cow’s milk allergy with a weekly up-dosing regimen: a randomized single-blind controlled study. Ann Allergy Asthma Immunol.

[24] Longo G, Barbi E, Berti I, Meneghetti R, Pittalis A, Ronfani L, et al (2008). Specific oral tolerance induction in children with very severe cow’s milk-induced reactions. J Allergy Clin Immunol.

[25] Kaneko H, Teramoto T, Kondo M, Morita H, Ohnishi H, Orji K, et al (2010). Efficacy of the slow dose-up method for specific oral tolerance induction in children with cow’s milk allergy: comparison with reported protocols. J Investig Allergol Clin Immunol.

[26] Brozek JL, Terracciano L, Hsu J, Kreis J, Compalati E, Santesso N, et al (2011). Oral immunotherapy for IgE-mediated cow’s milk allergy: a systematic review and meta-analysis. Clin Exp Allergy.

[27] Savilahti EM, Kuitunen M, Valori M, Rantanen V, Bardina L, Gimenez G, et al (2014). Use of IgE and IgG4 epitope binding to predict the outcome of oral immunotherapy in cow's milk allergy. Pediatr Allergy Immunol.

[28] Jagdis A, Berlin N, Barron C, Giruparajah M, Leader N, Maclachlan S, et al (2014). Effect of ketotifen premedication on adverse reactions during peanut oral immunotherapy. Allergy Asthma Clin Immunol.

[29] Sanchez-Garcia S, Rodriquez del Rio P, Escudero C, Garcia-Fernandez C, Ramirez A, Ibanez MD (2012). Efficacy of oral immunotherapy protocol for specific oral tolerance induction in children with cow’s milk allergy. Isr Med Assoc J.

[30] Alvaro M, Giner MT, Vazquez M, Lozano J, Dominguez O, Piquer M, et al (2012). Specific oral desensitization in children with IgE-mediated cow's milk allergy. Evolution in one year. Eur J Pediatr.

[31] Sato S, Yanagida N, Ogura K, Asaumi T, Okada Y, Koike Y, et al (2014). Immunotherapy in food allergy: towards new strategies. Asian Pac J Allergy Immunol.

[32] Buchanan AD, Green TD, Jones SM, Scurlock AM, Christie L, Althage KA, et al (2007). Egg oral immunotherapy in nonanaphylactic children with egg allergy. J Allergy Clin Immunol.

[33] Keet CA, Frischmeyer-Guerrerio PA, Thyagarajan A, Schroeder JT, Hamilton RG, Boden S, et al (2012). The safety and efficacy of sublingual and oral immunotherapy for milk allergy. J Allergy Clin Immunol.

